# Oncologic Benefit of Adjuvant Chemoradiation after D2 Gastrectomy: A Stepwise Hierarchical Pooled Analysis and Systematic Review

**DOI:** 10.3390/cancers12082125

**Published:** 2020-07-31

**Authors:** Chai Hong Rim, In-Soo Shin, Hye Yoon Lee, Won Sup Yoon, Sunmin Park

**Affiliations:** 1 Department of Radiation Oncology, Ansan Hospital, Korea University Medical College, Gyeonggido 15355, Korea; irionyws@korea.ac.kr (W.S.Y.); sunmini815@gmail.com (S.P.); 2 Graduate school of Education, Dongguk University, Seoul 04620, Korea; s9065031@dongguk.edu; 3 Department of General Surgery, Ansan Hospital, Korea University Medical College, Ansan, Gyeonggido 15355, Korea; heygemma@korea.ac.kr

**Keywords:** D2 gastrectomy, stomach cancer, gastric cancer, chemoradiotherapy, radiation therapy

## Abstract

Our study aimed to evaluate the benefits of chemoradiotherapy (CRT) after D2 gastrectomy, as compared to adjuvant chemotherapy, alone. PubMed, MEDLINE, Embase, and the Cochrane Library were systematically searched. We applied stepwise analyses that enabled the evaluation of data from randomized controlled trials (RCTs), balanced studies, and all studies separately and in a hierarchical manner. Thirteen controlled studies, including six RCTs involving 2603 patients, were included. Overall pooled analysis revealed a disease-free survival benefit of CRT (odds ratio (OR): 1.264, *p* = 0.053), which was more evident in the subgroup analysis of RCTs (OR: 1.440, *p* = 0.006) and balanced studies (OR: 1.417, *p* < 0.001). Overall survival was insignificantly different in the overall pooled analysis (OR: 1.124, *p* = 0.347). However, the difference was marginally significant in the subgroup analysis of balanced studies (OR: 1.279, *p* = 0.055) and significant in the subgroup analysis of studies involving stage ≥III patients only (OR: 1.663, *p* = 0.005). Locoregional recurrence (LRR) reduction was noted in the overall pooled analysis (OR: 0.559, *p* = 0.012; pooled rate: 11.3% vs. 18.1%) and was more robust in the subgroup analyses. Grade ≥3 leukopenia was higher in the CRT arm (OR: 1.387, *p* = 0.004; pooled rate: 26.4% vs. 15.7%). CRT after D2 gastrectomy should be applied for patients with high risk of LRR (e.g., stage ≥ III), along with efforts to reduce leukopenia.

## 1. Introduction

Gastric cancer is responsible for up to 800,000 deaths annually and is the third leading cause of cancer-related death [1]. The mainstay curative modality for gastric cancer is surgical resection. Except for early-stage cases that are amenable to endoscopic resection (e.g., well-differentiated tumors <2 cm in size), gastrectomy with D2 lymphatic dissection (D2 gastrectomy) is commonly applied, specifically in Asian countries [2,3,4,5,6]. Adjuvant chemotherapy (CT) was previously found to benefit western patients, in whom D2 gastrectomy is less commonly performed [7,8]. Recent landmark randomized controlled trials (RCTs) confirmed the oncologic benefit of S-1 or capecitabine plus oxaliplatin for Asian patients; hence, adjuvant CT is widely administered to these patients (including those who underwent D2 gastrectomy) [9,10].

Although the Intergroup-0116 trial revealed the benefit of adjuvant chemoradiotherapy (CRT) [11], it was heavily criticized for its insufficient extent of surgery, as fewer than 10% of patients underwent D2 gastrectomy. Therefore, a large tertiary center in South Korea conducted an RCT that compared the benefit of CRT to that of CT in patients who underwent D2 gastrectomy. This “Adjuvant Chemoradiation Therapy in Stomach Cancer” (ARTIST) trial failed to meet its primary endpoint of disease-free survival (DFS, *p* = 0.0862) [12]. Therefore, adjuvant CRT is generally not recommended after D2 gastrectomy in major clinical guidelines except for high-risk subgroups with R1 resection or remnant disease [2,3,4,13,14,15].

Locoregional recurrence (LRR) remains a burden even after D2 gastrectomy. In a previous large surgical series of up to 1500 patients who underwent D2 gastrectomy [16], grossly one-quarter experienced recurrences, approximately one-third (32.5%) of which were locoregional. The survival period for patients with LRR was only 1.5 months longer than that for patients with hematogenous metastases. Although the primary endpoint was not reached in the ARTIST trial, LRR was significantly reduced (7% vs. 13%, *p* = 0.0033) and DFS was extended following CRT in the subgroup of patients with lymphatic metastases [17]. Several other clinical trials also revealed LRR and DFS benefits for CRT, which reflected real-world clinical experiences and practices [18,19,20,21]. Furthermore, recent technological advancements in radiation therapy have gradually lowered the risk of possible complications by reducing bystander irradiation to the organs-at-risk [22,23,24].

Our study aimed to integrate and analyze data from controlled trials that compared CRT and CT for patients with gastric cancer who underwent D2 gastrectomy and to thereby evaluate the practical role of CRT using real-world studies and RCTs.

## 2. Results

**Figure 1:**
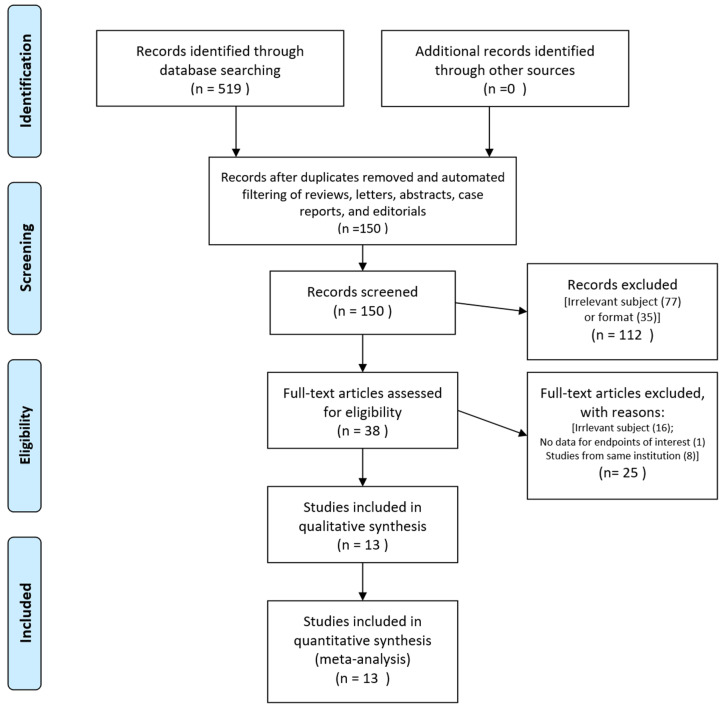
Flowchart of the study selection process.

Thirteen studies [17,18,19,20,21,25,26,27,28,29,30,31,32] involving 2603 patients with gastric cancer who underwent D2 resection were included (Figure 1). Six studies were RCTs [17,18,21,26,29,30] and seven were non-RCTs [19,20,25,27,28,31,32]; nine studies were considered balanced between their two arms [17,18,19,20,21,29,30,31,32]. The studies’ quality scores are shown in Table S1; all studies included patients who achieved R0 resection after D2 gastrectomy except one in which approximately one-quarter of the patients had undergone R1 resection [27]. The studies’ backgrounds and clinical information are shown in Table 1 and Table 2, respectively.

The pooled odds ratio (OR) for DFS was 1.264 (95% confidence interval (CI): 0.997–1.603, *p* = 0.053) favoring the CRT arm with a medium degree of heterogeneity (*p* = 0.1, I^2^ = 37.5%). In the subgroup analyses of RCTs alone, the pooled OR increased to 1.440 (95% CI: 1.110–1.867, *p* = 0.006), and heterogeneity was significantly diminished (*p* = 0.964, I^2^ = ~0.0%). Subgroup analysis of only the balanced studies showed similar results, with an OR of 1.417 (95% CI: 1.171–1.715, *p* < 0.001) and very low heterogeneity (*p* = 0.61, I^2^ = ~0.0%). In single-arm analyses, the 3-year DFS rates were 62.5% (95% CI: 54.6–69.8) and 57.1% (95% CI: 46.1–67.5) in the CRT and CT arms, respectively; the corresponding 5-year DFS rates were 53.3% (95% CI: 40.9–56.4) and 40.9% (95% CI: 27.3–56.0), respectively. The heterogeneity among studies in the single-arm analyses was very high and significant.

**Figure 2:**
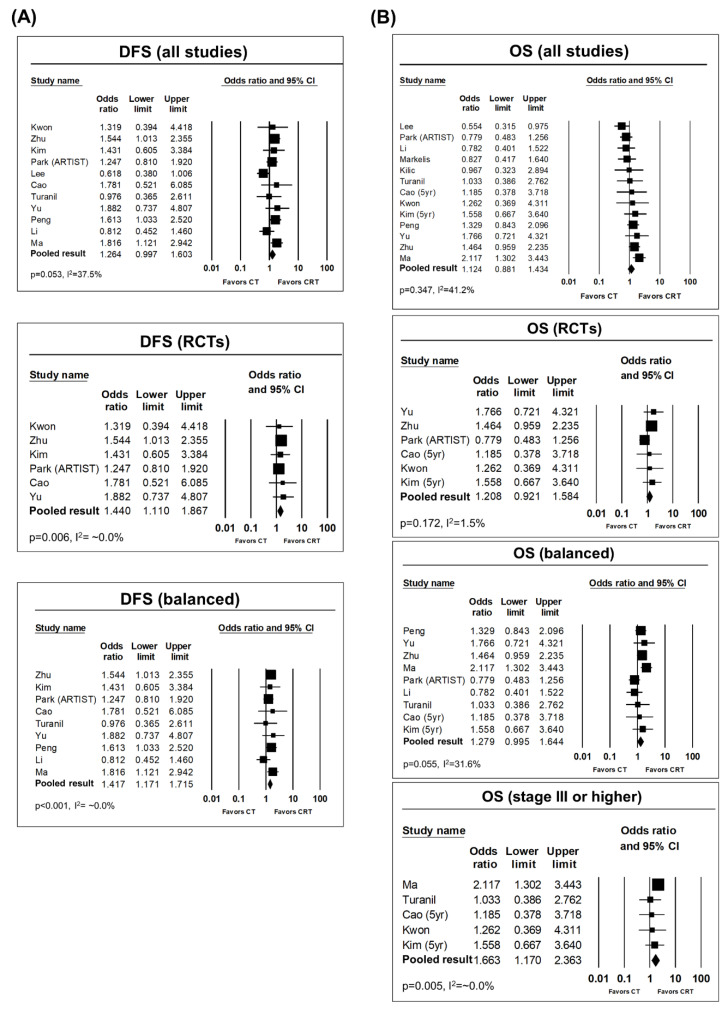
(**A**) Forest plots of overall and subgroup pooled analyses of 3-year disease-free survival (DFS). (**B**) Forest plots of overall and subgroup pooled analyses of 3-year overall survival (OS). CI, confidence interval; RCT, randomized controlled trial.

The overall pooled OR for overall survival (OS) was 1.124 (95% CI: 0.881–1.434, *p* = 0.347). The pooled ORs of subgroup analyses of RCTs and the balanced studies were 1.208 (95% CI: 0.921–1.584, *p* = 0.172) and 1.279 (95% CI: 0.995–1.644, *p* = 0.055), respectively. A medium degree of heterogeneity was found in overall pooled analysis and subgroup analysis of the balanced studies, and the subgroup analysis of RCTs alone had very low heterogeneity. Considering the marginally significant result in the subgroup analysis of RCTs alone, in a further subgroup analysis of studies involving stage ≥III patients only, the pooled result was statistically significant with an OR of 1.663 (95% CI: 1.170–2.363, *p* = 0.005). The pooled 3-year OS rates in the single-arm analyses were 61.4% (95% CI: 51.1–70.8) and 59.1% (95% CI: 44.4–72.3) in the CRT and CT arms, respectively; the corresponding 5-year OS rates were 55.8% (95% CI: 44.5–66.5) and 46.9% (95% CI: 32.0–62.4), respectively. The heterogeneity among studies was very high and significant on single-arm analysis. Prognosticators of OS and DFS were available in four and two studies, respectively. Regarding OS prognosticators, cancer stage was significant in three of the four studies, and positive lymph nodes in surgical pathology or a high lymph node ratio (i.e., number of positive lymph nodes/total dissected lymph nodes) was significant in all four studies. Among DFS prognosticators, the lymph node ratio was significant in one study, and cancer stage and age were significant in the other study. Forest plots of the pooled analyses of 3-year DFS and OS rates are shown in Figure 2.

**Figure 3:**
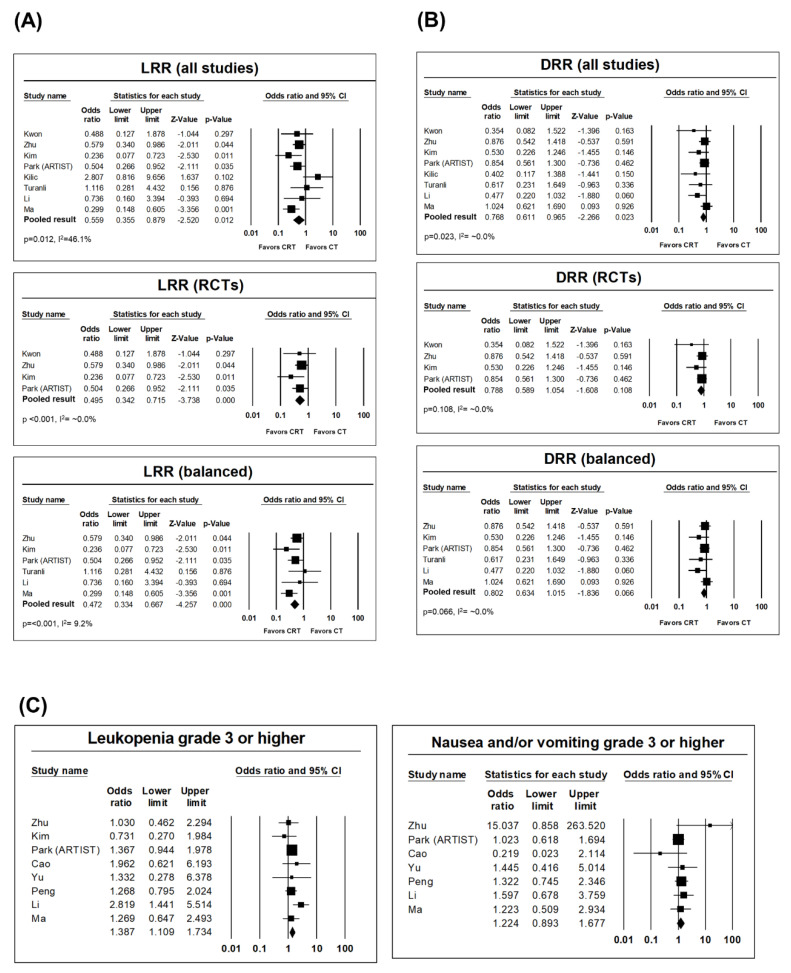
(**A**) Forest plots of overall and subgroup pooled analyses of locoregional recurrence rate (LRR). (**B**) Forest plots of overall and subgroup pooled analyses of distant recurrence rate (DRR). CI, confidence interval; RCT, randomized controlled trial. (**C**) Forest plots of pooled analyses of leukopenia and nausea and/or vomiting.

The LRR rates were significantly lower in the CRT arms than in the CR arms. The pooled OR was 0.559 (95% CI: 0.355–0.879, *p* = 0.012) for all studies, with a medium degree of heterogeneity (*p* = 0.073, I^2^ = 46.1%). In subgroup analyses of RCTs, the OR was 0.495 (95% CI: 0.342–0.715, *p* < 0.001) with very low heterogeneity (*p* = 0.569, I^2^ = ~0.0%). In the balanced studies, the OR was 0.472 (95% CI: 0.334–0.667) with a low degree of heterogeneity (*p* = 0.357, I^2^ = 9.2%). Single-arm analyses revealed overall pooled LRR rates of 11.3% (95% CI: 7.5–1.68) and 18.1% (95% CI: 13.1–24.4) in the CRT and CR arms, respectively. As for the distant recurrence rate (DRR), the OR was 0.768 (95% CI: 0.611–0.965, *p* = 0.023) favoring the CRT arm, with very low heterogeneity (*p* = 0.504, I^2^ = ~0.0%). However, there was no statistically significant difference in the subgroup analysis of RCTs alone (OR: 0.788, 95% CI: 0.589–1.054, *p* = 0.108), whereas borderline significance was noted when analyzing balanced studies alone (OR: 0.802, 95% CI: 0.634–1.015, *p* = 0.066). DRR rates on pooled single-arm analyses were 24.1% (95% CI: 18.1–31.2) and 30.9% (95% CI: 24.4–38.3) in the CRT and CT arms, respectively. Heterogeneities were high and significant on single-arm pooled analyses of LRR and DRR (Figure 3A,B).

The most common grade ≥3 complications reported were leukopenia and nausea and/or vomiting. Leukopenia was reportedly higher in the CRT arm than in the CR arm, with a pooled OR of 1.387 (95% CI: 1.109–1.734, *p* = 0.004). Nausea and/or vomiting was not significantly different between the two arms, with an OR of 1.224 (95% CI: 0.893–1.677, *p* = 0.209). Heterogeneity was very low in both the abovementioned analyses. In single-arm pooled analyses, the incidence rates of leukopenia were 26.4% (95% CI: 17.7–37.5) and 15.7% (95% CI: 9.4–25.0) in CRT and CT arms, respectively, and the incidence rates of nausea and/or vomiting were 11.2% (95% CI: 7.8–15.8) and 11.1% (95% CI: 7.6–16.0) in CRT and CT arms, respectively. Heterogeneity was high in both single-arm analyses (Figure 3C). Regarding grade 5 toxicity, one fatal complication was found in each arm of the pooled studies.

Possible publication bias was observed in the overall pooled analysis of DRR, the subgroup analysis of OS of studies involving stage ≥ III patients only, and single-arm analyses of leukopenia and nausea and/or vomiting. Sensitivity analyses using Duval and Tweedie’s method revealed that, in the overall pooled analysis of DRR, the OR was mildly increased, and the statistical significance of the difference was decreased (trimmed OR: 0.837, 95% CI: 0.655–1.070). Regarding the subgroup analysis of OS with studies involving stage ≥III patients only, trimmed OR was increased to 1.963, which reflects a more significant result. The results of all the pooled analyses are detailed in Table 3.

## 3. Discussion

### 3.1. Clinical Interpretation of Pooled Results and Related Literature

Gastric cancer has a tendency to spread locoregionally and hematogenously, even after an extended surgical approach [16]. The addition of radiotherapy to neoadjuvant or adjuvant CT could further reduce LRR by eradicating subclinical tumor foci in the perigastric region. CRT has been applied in both neoadjuvant and adjuvant settings. The German POET (Preoperative chemotherapy versus chemoradiotherapy in locally advanced adenocarcinomas of the oesophagogastric junction) trial reported a higher pathologic complete remission and node-negative status and extended OS (3-year: 47.4% vs. 27.7%) compared with neoadjuvant CT alone [33]. Another RCT from Nordic countries reported results with similar trends, with higher pathologic complete remission, lymph node negative status, and R0 status [34]. Although these results were significant, most of the relevant studies were conducted on patients who were diagnosed with diseases in the esophagus or gastroesophageal junction. Therefore, extrapolating these results to other populations, specifically Asians, who are generally diagnosed with stomach cancer that is mainly observed in the body or non-cardia area, might be irrelevant [3,35]. Considering its high incidence in Asia and southern Europe, several clinical guidelines recommend upfront D2 gastrectomy for cases, except in very early disease (e.g., T1N0), and discuss the necessity of adjuvant treatment including CT and CRT [2,3,4,15].

Our study’s first take-home message is that the application of CRT after D2 resection has a clear oncologic benefit in terms of DFS and LRR. The more significant results in the subgroup analyses with markedly lower heterogeneity reflect a clearer oncologic benefit with better clinical distribution between the two arms. Considering the difference in DFS rates between the CRT and CT arms, which was wider at 5 years (53.3% vs. 40.9%) than at 3 years (62.5% vs. 57.1%) on single-arm analysis, mid- to long-term locoregional control with CRT may have contributed to a longer DFS. In a previous surgical series, locoregional and hematogenous recurrences occurred in 32.5% and 34.3% of ~1500 patients, respectively, who underwent D2 gastrectomy and experienced subsequent failure; their corresponding mean survival periods were similar (11.0 and 9.4 months, respectively) [16]. Patients in that study who underwent curative resection for LRR had a mean survival of 21.6 months. These results demonstrate that locoregional and hematogenous recurrences markedly impact oncologic outcomes, indicating that longer survival might be achieved by reducing LRR. Furthermore, Chang et al. [36]. reported that the most prevalent nodal recurrences among patients with stage III gastric cancer were outside the D2 dissection field; hence, adjuvant radiotherapy might control recurrences in those nodal basins.

Although DRR decreased according to the overall pooled analysis, these findings were not significant in the subgroup analysis with RCTs alone. Cancers tend to occur step-wise, developing locally in the primary site and ultimately spreading systemically [37]. Controlling LRR might affect DRR according to previous studies of other malignancies [38,39], but data regarding gastric cancer are insufficient. The possible correlation between LRR reduction via CRT and improved DRR should be further investigated.

Although DFS was shown to be an acceptable surrogate for OS in gastric cancer [40], improvements in DFS and LRR might be insufficient to significantly alter common practice and overcome concerns regarding additional toxicities. The next step for optimizing CRT for gastric cancer is to identify patients who can most benefit from LRR reduction and ultimately experience longer OS. Peng et al. [31] reported improved OS with CRT compared with CT in their subgroup of patients with stage IIIC disease (median OS: 29 vs. 23 months, *p* = 0.049). A study by Ma et al. [19], which was one of the largest series performed, revealed that CRT provided an OS benefit over CT among patients with stage IIIA and IIIB disease (the 5-year OS rates for stage IIIA patients were 61.5% vs. 34.5% *p* = 0.03; those for IIIB patients were 46.4% vs. 26.1%, *p* = 0.035). Although the result was not significant in overall pooled analysis, the subgroup analysis of studies involving stage ≥III patients only showed a significant benefit regarding OS (OR 1.663, *p* = 0.005), suggesting selective application of CRT after D2 gastrectomy for patients with locally advanced diseases.

Regarding complications, the most problematic complication in response to radiation added to CT was leukopenia. Since hematopoietic cells are highly fragile to radiation exposure, reducing bystander irradiation with the application of intensity-modulated radiotherapy (IMRT) can reduce this complication [41,42]. Specific bone marrow-sparing technique can also be helpful in reducing leukopenia [43]. Of note, the study by Zhu et al. [18] has reported a significantly low rate of leukopenia (7.5%) after CRT using IMRT and has shown a favorable survival in the CRT arm (median OS: 54 vs. 38 months, *p* = 0.122). Furthermore, CRT should be carefully performed for patients with compromised immunity, such as those having malignancy related with common variable immunodeficiency [44,45].

A limitation inherent to literature analysis is that the majority of studies are published in only a few countries in East Asia. Considering that gastric cancer is the third leading cause of cancer-related death globally, the literature remains significantly insufficient. Hence, with respect to administering CRT after D2 gastrectomy, major clinical guidelines depend almost entirely on data from the ARTIST trial [3,14,15], as guidelines from Japan [2] and Italy [4] do not even mention radiotherapy and CRT as possible modalities after D2 gastrectomy. Unfortunately, the ARTIST trial marginally failed to achieve the primary DFS endpoint (*p* = 0.0862) [12]; this result led to highly conservative recommendations of CRT after D2 resection in the guidelines, such as only for patients with remnant disease (R1 or R2 section) or resection with less-extensive lymphadenectomy (D0 or D1). However, as stated by the American Statistical Association, binary decisions solely depend on a predefined *p*-value that might cause serious misinterpretation [46]. Furthermore, the clinical significance of subgroup analyses and those of the entire targeted population should also be emphasized. In the ARTIST trial, subgroup analysis of N+ patients showed a significant DFS benefit (*p* = 0.0365), and nearly 90% of the patients belonged to various N+ subgroups. When considering this, the ARTIST trial may not have actually “failed” as deemed in several clinical guidelines [2,3,4,14,15]. Moreover, although complications tended to increase in the CRT arms of the present meta-analysis, most were transient, and grade 5 complications were significantly rare. Wider application of modern radiotherapy techniques is expected to diminish toxicities, as mentioned above [41,42,43]. Taken altogether, the application of CRT after D2 gastrectomy should be considered for patients with high-risk of LRR, and future randomized studies should identify specific subgroups that can derive OS benefit from CRT, prompted by locoregional benefits and the application of modern techniques.

### 3.2. Practical Implication and Future Perspectives

Adjuvant CRT after D2 gastrectomy is not commonly practiced in clinical settings, despite its benefits in locoregional control [3]. Although our results cannot change current clinical practice in a short time, our study might at least stimulate the conduction of clinical trials applying adjuvant CRT, specifically for advanced cases with stage ≥III. Multidisciplinary approach has been more rarely performed for gastric cancer in clinical settings compared with other cancers. However, it was recently reported that the diagnoses of gastric cancer were changed in 18–27% of patients, and 23–77% of treatment policies were changed after multidisciplinary discussion [47,48,49]. The results of our study will further increase the necessity of multidisciplinary discussions to optimize clinical decision.

Several molecular pathways are known to be involved in gastric carcinogenesis, such as human epidermal growth factor receptor 2 (HER2), HER3, epidermal growth factor receptor (EGFR), hepatocyte growth factor receptor/c-MET, E-cadherin, matrix metalloproteinase, vascular endothelial growth factor (VEGF)/VEGF receptor (VEGFR), WNT/β-catenin, fibroblast growth factor receptor, and Akt/PI3K/mTOR, in gastric cancer and other gastrointestinal (GI) malignancies [50]. Additional administration of trastuzumab, a recombinant monoclonal antibody against HER2, has shown a better significant survival benefit compared with conventional CT alone [51]. A monoclonal antibody targeting VEGFR-2, ramucirumab, has also shown significant benefit as a second-line systemic treatment agent [52,53]. Novel molecular findings regarding drug resistance and regional dissemination of GI cancers will stimulate the development of future systemic agents to overcome these clinical hindrances [54,55]. So far, CRT has been only studied as a radiotherapy combined with conventional CT, either in a neoadjuvant or adjuvant setting [3,13]. The results of the present study will encourage future studies optimizing gastric cancer treatment by combining radiotherapy with target agents or newer agents actively being researched.

### 3.3. Limitations

A limitation of this meta-analysis was that the overall numbers of studies and of RCTs with a sufficient number of enrolled patients were small. A meta-analysis increases the sample size and, consequently, the power to study the effects of interest by combining primary studies while considering the sizes of the studies included. However, one number cannot summarize a research field and can be affected by the heterogeneity of studies, by publication bias and by the fact that not all variables are comparable despite the performance of complementing statistical methods. Clinical application of meta-analysis results should be based on the interpretation of both pooled data and detailed information from individual studies that reflect actual clinical practices [56]. Meta-analyses of non-randomized controlled studies (NRCTs) have been controversial because their potential heterogeneity might skew the pooled analyses; however, oncology research cannot always derive the firmest evidence from RCTs, and carefully performed meta-analyses including NRCTs might provide clinically useful information in obscure areas [57]. Previous systematic reviews also found that meta-analyses based on RCTs versus high-quality studies had similar outcome estimates [58]. Compared with previous meta-analyses of related subjects [59,60,61], our study included the largest number of RCTs, and patients were analyzed from real-world data and NRCTs with no language restrictions during searching. Furthermore, by performing sensitivity analyses (including RCTs and balanced studies), we attempted to minimize the potential bias of including NRCTs and to improve the reliability of outcomes involving real-world data.

## 4. Methods

This study was designed to answer the PICO question “is there an oncologic benefit of adjuvant CRT compared to CT after D2 resection for gastric cancer in a real-world clinical setting?” and adhered to the Preferred Reporting Items for Systematic Reviews and Meta-Analyses guidelines. A systematic search of the PubMed, MEDLINE, Embase, and Cochrane Library databases was performed up to 20 January 2020, using the following search terms: “(gastric OR stomach) AND cancer AND (radiotherapy OR ‘radiation therapy’) AND survival AND D2.” No language or publishing period restriction was applied. Full-text publications were searched to identify a rational clinical comparison between the CRT and CT arms.

### 4.1. Inclusion Process and Criteria

The primary endpoint of the study was DFS and OS. The secondary endpoints included LRR, DRR, and grade ≥ 3 complication rates. Our inclusion criteria were as follows: (1) controlled clinical trials aimed at comparing CRT and CT after D2 resection for gastric cancer, (2) inclusion of at least 10 patients in each arm, and (3) reporting of at least one survival outcome (DFS or OS).

After the initial search, duplicate studies, conference abstracts, reviews, letters, editorials, case reports, laboratory studies, and irrelevant studies were filtered out using titles and citations. The remaining studies were assessed by reviewing their abstracts and full texts to identify those that met all of the inclusion criteria. We included multiple studies from the same institutions only if they did not have overlapping populations or if any overlap was negligible. For studies with possibly overlapping populations, we selected only one using the following criteria (prioritized in numerical order): (1) the study with a higher level of evidence (e.g., RCTs were preferred over NRCTs), (2) that with the larger number of patients, and (3) the most recently published. All studies were selected by two independent researchers who resolved any disagreements by mutual discussion.

### 4.2. Data Collection and Quality Assessment

Data acquisition was performed by two independent researchers using a pre-standardized form, including background information of authors, affiliations, study design, patients recruiting periods, and number of patients; clinical information including treatment modality, patients’ age, rate of diffuse type cancer cases, and T and N stages; clinical outcomes including DFS, OS, prognosticators of survival outcomes, pattern of failure, and grade 3 or higher toxicities. Missing DFS and OS rates were estimated from the descriptive graphs in consideration of the follow-up periods. For quality assessment of the included NRCTs and RCTs, the Newcastle–Ottawa scale was used [62]. Studies with scores of ≥8 were considered high quality, scores of 7–8 medium quality, and the remainder low quality.

### 4.3. Statistical Analyses

Considering the range of clinical diversity, the different institutions with distinct treatment modalities, and the inclusion of studies (NRCTs and RCTs) of different designs, a random effects model was used for the pooled analysis of endpoints [63]. We performed pooled analyses in a stepwise-hierarchical manner; ORs calculated from the comparison of endpoints between the CRT and CT arms were pooled and analyzed for all studies, RCTs alone, and balanced studies alone. Balanced studies were defined as those without significant differences (*p* < 0.05) in the patients’ clinical profiles (i.e., age, histologic type, and TNM stage). Heterogeneity among studies was evaluated using I^2^ statistics [64] and the Cochran Q test [65]. Significant heterogeneity was considered present when *p* < 0.1 and I^2^ ≥ 50%; I^2^ values of 25%, 50%, and 75% corresponded to low, moderate, and high degrees of heterogeneity, respectively [66]. Visual inspection of funnel plots and the Egger’s test [67] were used to identify possible publication biases. For pooled analyses with significant asymmetry in funnel plots or two-tailed *p*-values < 0.1 in the Egger’s test, Duval and Tweedie’s trim and fill method [68] was performed for sensitivity analysis. All statistical analyses were designed and confirmed by both a clinician and a biostatistician specialized in meta-analysis and performed using Comprehensive Meta-Analysis version 3 (Biostat Inc., Englewood, NJ, USA).

### 4.4. Ethical Consideration

Ethical approval was not required because this study retrieved and synthesized data from already published studies. Otherwise, authors declare that the investigations were performed following the principles of the Declaration of Helsinki of 1975 [69], revised in 2013.

## 5. Conclusions

Our study clearly demonstrated the benefits of CRT after D2 gastrectomy in terms of DFS and LRR and also a possibility of decreased DRR. Although the result was not significant in overall pooled analysis, OS benefit was shown in the subgroup analysis of studies involving stage ≥ III patients only. Therefore, CRT after D2 gastrectomy should be applied for selected patients with a high risk of LRR, such as those with stage ≥ III disease, along with technical efforts such as IMRT or bone marrow-sparing technique, to reduce complications including leukopenia. Future randomized studies should focus on identifying specific subgroups of patients who can benefit from CRT after D2 gastrectomy considering OS.

## Figures and Tables

**Table 1:** Background information of patients in the included studies.

Author, Year	Affiliation	Study Design	Inclusion Criteria	Inclusion Period	Total No. of Patients	CRT/CT No.	Modality	^†^ Age (Years)	Diffuse Type (%)	T Stage	N Stage
Markelis, 2009 [25]	Kaunas University, Lithuania	NRCT, prospective	Radical resection and D2 LND I–IV	2003–2007	133	63	FL, 45 Gy/5 weeks	m57.9	HG 3–4 (78%)	T1–2 (30%); T3–4 (70%)	N0 (29%); N1 (41%); N2 (30%)
						70	5FU and LV	m62.2	HG 3–4 (63%)	T1–2 (33%); T3–4 (67%)	N0 (27%); N1 (44%); N2 (29%)
							*p*-value	**0.017**	0.0921	0.883	0.685
Kwon, 2010 [26]	Dong-A University, South Korea	RCT	R0 resection and D2 LND III–IV	2002–2004	61	31	FP and capecitabine, 45 Gy/5 weeks	≥60 (25.8%)	64.5	AJCC IIIA (36%); IIIB (42%); IV (M0, 23%)	
						30	FP	≥60 (46.7%)	43.3	AJCC IIIA (67%); IIIB (23%); IV (M0, 10%)	
							*p*-value	0.114	0.108	**0.05**	
Zhu, 2012 [18]	Nanjing Medical University, China	RCT	R0 resection and D2 LND IB–IV	2003–2008	351	186	FL and 45 Gy/5 weeks (IMRT)	M56	NA	AJCC IB–II (30%); III (55%); IV (M0, 14%)	
						165	5FU and LV	M59	NA	AJCC IB–II (27%); III (58%); IV (M0, 14%)	
							*p*-value	NA			
Kim, 2012 [21]	NCC, Korea	RCT	R0 resection and D2 LND stage III–IV	2002–2006	110	46	FL and 45 Gy/5 weeks	≥60 (19.6%)	56.5	T2 (30%); T3 (63%); T4 (7%)	N1 (33%); N2 (46%); N3 (22%)
						44	FL	≥60 (31.8%)	54.5	T2 (43%) T3 (50%); T4 (7%)	N0–1 (27%); N2 (50%); N3 (23%)
							*p*-value	0.23	0.978	0.465	0.483
Park, 2015, Lee, 2012 (ARTIST) [12,17]	Samsung Medical Center, Korea	RCT	R0 resection and D2 LND IB–IV	2004–2008	458	230	XP and 45 Gy/5 weeks	M56	63%	AJCC II (37%); III (31%); IV(M0, 11%) N0 (12%); N1 (57%); N2–3 (32%)	
						228	XP	M56	57%	AJCC II (38%); III (29%); IV (12%) N0 (15%); N1 (54%); N2–3 (31%)	
							*p*-value	NA			
Kilic, 2013 [27]	Istanbul University, Turkey	NRCT, retrospective	D2 LND, pTanyN3M0 (R0 77.8% vs. 71.7%, *p* = 0.61)	2005–2009	71	18	FL and 45 Gy/5 weeks	M46	61.1	T2/3 (89%) T4 (11%)	all N3
						53	ECF of DCF	M54	30.1	T2/3 (91%) T4 (9%)	all N3
							*p*-value	0.1	**0.02**	0.72	
Lee, 2014 [28]	Samsung Medical Center, Korea	NRCT, retrospective	R0 resection and D2 LND IB–IV	2008–2009	405	244	FL and 45 Gy/5 weeks	M53	Tubular (68.4%) signet ring cell (22.1%)	T1–2 (71%) T3–4 (29%)	N0–2 (56%) N3a (27%) N3b (17%)
						161	S-1	M57	Tubular (67.1%) signet ring cell (21.1%)	T1–2 (82%) T3–4 (18%)	N0–2 (78%) N3a (17%) N3b (5%)
							*p*-value	**0.001**	0.234	**0.02**	**<0.001**
Cao, 2015 [29]	Xinwen Mining Group Central Hospital, China	RCT	R0 resection and D2 LND stage III–IV	2008–2010	50	25	FP and capecitabine CCRT	≥60 (40%)	LD (68%)	AJCC IIIA (32%); IIIB (40%)	
						25	FP	≥60 (44%)	LD (60%)	AJCC IIIA (48%); IIIB (32%)	
							*p*-value	NS	NS	NS	
Turanli, 2015 [20]	Ankara Oncology Education and Research Hospital, Turkey	NRCT, retrospective	R0 resection and D2 LND III	2004–2009	92	71	FL and 45 Gy/5 weeks	m57.5	29.6	T3 (7%) T4 (89%)	N1 (18%); N2 (40%) N3 (42%)
						21	FL or ECF	m57.5	38.1	T3 (14%) T4 (81%)	N1 (14%); N2 (38%); N3 (47%)
							*p*-value	0.98	0.46	0.48	0.87
Yu, 2016 [30]	Anhui University, China	RCT	R0 resection and D2 LND stage II–III	2010–2011	79	40	Capecitabine CCRT (45 Gy) followed by XELOX	≥60 (57.5%)	HG3–4 (57.5%)	AJCC II (40%) III (60%) N0 (29%)	
						39	XELOX	≥60 (48.7%)	HG3–4 (64.1%)	AJCC II (36%) III (64%) N0 (31%)	
							*p*-value	0.434	0.548	0.707	
Peng, 2016 [31]	Wuhan University, China	NRCT, retrospective	R0 resection and D2 LND stage IIA–IIIC	2004–2012	337	124	FOLFOX or XELOX or capecitabine and 45 Gy/ 5 weeks	M54	HG 3–4 (78.2%)	AJCC II (36%); IIIA (20%); IIIB (17%) IIIC (27%)	
						213	FOLFOX or XELOX or capecitabine	M56	HG 3–4 (75.5%)	AJCC II (31%); IIIA (23%); IIIB (20%); IIIC (27%)	
							*p*-value	0.247	0.128	0.507	
Li, 2017 [32]	Fudan University, China	NRCT, retrospective	R0 resection and D2 LND IB–IIIC	2005–2010	186	93	5FU or capecitabine or tegafur and 45–50Gy/5–6 weeks (3D-CRT or IMRT)	m54	LD (15.1%)	T1–2 (17%) T3 (25%) T4 (58%)	N0 (9%); N1 (17%); N2 (23%); N3 (52%)
						93	5FU based regimen	m57	LD (19.4%)	T1–2 (14%) T3 (19%) T4 (67%)	N0 (11%); N1 (22%); N2 (24%); N3 (44%)
							*p*-value	0.61	0.43	0.37	0.76
Ma, 2019 [19]	Fudan University, China	NRCT, retrospective, PSM	R0 resection and D2 LND stage III	2009–2014	270	135	Same regimen and 45 Gy/5 weeks, 5–10 Gy boost (3D-CRT or IMRT)	m54.3	NA	T2 (6%) T3 (16%) T4 (79%)	N0–2 (31%) N3a (45%) N3b (24%)
						135	5FU or capecitabine or ECF, modified DCF regimen	m54.7	NA	T2 (3%) T3 (21%) T4 (76%)	N0–2 (30%) N3a (44%) N3b (25%)
							*p*-value	0.74		0.305	0.081

Abbreviations: CRT, chemoradiation; CCRT, concurrent chemoradiation; CT, chemotherapy; RCT, randomized controlled trial; LND, lymph node dissection; 5FU, 5-fluorouracil; FL, 5-fluorouracil and leucovorin; FP, 5-fluorouracil plus cisplatin; IMRT, intensity-modulated radiotherapy; NA, not assessed; NCC, National Cancer Center; XP, capecitabine and cisplatin; AJCC, American Joint Committee on Cancer; ECF, epirubicin, cisplatin, fluorouracil; DCF, docetaxel, cisplatin, fluorouracil; LD, low differentiation; NRCT, non-randomized controlled trials; XELOX, capecitabine and oxaliplatin; HG, high-grade; PSM, propensity score matching. ^†^ Upper case M denotes the median, and lower case m denotes the mean. Statistically significant *p*-values marked in bold font.

**Table 2:** Clinical outcomes of patients in the included studies.

Author, Year	CRT/CT No.	Median Follow-Up (Months, Range)	Median DFS	3-Year DFS	5-Year DFS	Median OS (Months)	3-Year OS	5-Year OS	Prognosticators (*p*-Value)	Pattern of Failure	Grade 3–4 Toxicities (CRT vs. CT)
Markelis, 2009 [25]	63						52.4%				Overall incidence: 44.4% vs. 7.1% (*p* < 0.05)
	70						57.1%				
							0.039				
Kwon, 2010 [26]	31	77.2 (24–92.8)		80.0%	76.7%		80.6%	70.1%		Overall LRR 12.9% vs. 23.3% (*p* = 0.335); DM 9.7% vs. 23.3% (*p* = 0.335)	Neutropenia (48.4% vs. 16.7%); anemia (12.9% vs. 16.7%); N/V (6.5% vs. 13.3%)
	30			75.2%	59.1%		76.7%	70.0%			
				0.887	0.222			0.814			
Zhu, 2012 [18]	186	42.5	50	57.5%	45.2%	54.0	59.7%	48.4%	UVA, OS: Stage (*p* < 0.001); LN+ (*p* = 0.001)	5-yr LRR 15.6% vs. 24.2% (CRT vs. CT, *p* = 0.042); 5-yr DM 24.2% vs. 26.7% (*p* = 0.595)	Leukopenia (7.5% vs. 7.3%); nausea (2.7% vs. 0%); vomiting (1.6% vs. 0%)
	165		32	46.7%	35.8%	38.0	50.3%	41.4%			
			**0.029**			0.122					
Kim, 2012 [21]	46	86.7		67.4%	60.9%			65.2%		Overall LRR 10.9% vs. 34.1% (CRT vs. CT, *p* = 0.006); Overall DM 32.6% vs. 47.7 (CRT vs. CT, *p* = 0.288)	Hematologic (19.6% vs. 25%, *p* = NS); GI toxicity (17.4% vs. 11.4%, *p* = NS).
	44			59.1%	50.0%			54.6%			
			0.246			0.67					
Park, 2015 Lee, 2012 (ARTIST) [12,17]	230	7 years		78.2%	73.9%		80.0%	75.0%	MVA, OS: Stage (*p* < 0.01); Lauren classification (*p* = 0.03); LNR (*p* < 0.01)	Overall LRR 7% vs. 13% (CRT vs. CT, *p* = 0.0033); DM 24% vs. 27% (*p* = 0.5568)	Neutropenia (48.4% vs. 40.7%); Nausea (12.3% vs. 12.4%); Vomiting (3.5% vs. 3.1%); One 5 complication in each arm
	228			74.2%	67.1%		83.7%	73.0%			
			0.0862			0.527					
Kilic, 2013 [27]	18	13.8 (6.2–74.1)	15.2			34.2	38.8%		MVA, DFS: LNR (*p* = 0.04)	Overall LRR 33.3% vs. 15.1% (CRT vs. CT, *p* = 0.63); DM 22.2% vs. 41.5% (CRT vs. CT)	No toxicity related death in both groups
	53		12.5			26.8	39.6%				
			0.56			0.74					
Lee, 2014 [28]	244	49 (3.0–62.0)		73.0%			79.8%		MVA, DFS: Stage (<0.001); Age (*p* = 0.006)		Neutropenia 40.2% vs. 8.7% (*p* < 0.001); all neutropenia was transient. Nausea 5.7% vs. 0% (*p* = 0.002); vomiting 2.5% vs. 0% (*p* = 0.085)
	161			81.4%			87.7%				
			**0.035**								
Cao, 2015 [29]	25	3 years		76.0%	60.0%			64.0%			Neutropenia 48% vs. 32% (*p* = 0.016); nausea 4% vs. 16% (*p* = 0.032); diarrhea 8% vs. 0% (*p* = 0.025)
	25			64.0%	52.0%			60.0%			
				0.112	0.231			0.324			
Turanli, 2015 [20]	71	30 (8–112)		42.2%	32.9%	32.0	43.6%	34.4%		Overall LRR 15.7% vs. 14.3% (CRT vs. CT, *p* = 0.089); DM 45.1% vs. 57.1% (*p* = 0.42)	
	21			42.8%	24.1%	29.0	42.8%	23.8%			
			0.8			0.74					
Yu, 2016 [30]	40	34		42.5%			52.5%		MVA, OS: ECOG (0.016); LN+ (0.035)		Leukopenia (10% vs. 7.7%, *p* = 0.253); N/V (17.5% vs. 12.8%, *p* = 0.043)
	39			28.2%			38.5%				
				0.238			0.235				
Peng, 2016 [31]	124	41.1 (14–111.1)	40.7	55.6%	38.7%	51.0	41.4%	45.6%			Overall incidence: 36.3% vs. 31.0% (*p* = 0.338) m/c Cx: Leukopenia or neutropenia (21.7% vs. 14.6%, *p* = 0.09); nausea (10.4% vs. 8.4%); vomiting (9.6% vs. 7.5%)
	213		31.2	43.7%	31.1%	48.6	34.7%	37.3%			
			0.112			0.3		0.132			
Li, 2017 [32]	93	CRT 28 (5–62) CT 43 (2–63)		57.0%			72.8%			Overall LRR 3.2% vs. 4.3% (*p* = 0.76); DM 12.9% vs. 23.7% (*p* = 0.18)	Overall incidence: 38.7% vs. 18.3% (*p* = 0.002); leukopenia 30.1% vs. 10.8%, Nausea (10.8% vs. 5.4%); vomiting (5.4% vs. 5.4%)
	93			62.0%			77.4%				
			0.3			0.23					
Ma, 2019 [19]	135	41 (7–104.2)		60.7%	40.7%	M51.2	57.0%	45.2%	MVA, OS: Stage (<0.001); LNR <0.001; total vs. subtotal gastrectomy 0.007); tumor deposit (0.028)	Overall LRR [*n* = 135 (CRT) 280 (CTx)] 7.4% vs. 21.1% (CRT vs. CT, *p* < 0.001); Overall DM 21.5% vs. 21.1% (CRT vs. CT, *p* = 0.924)	Leukopenia (16.3% vs. 13.3%, *p* = 0.493); anorexia (14.8% vs. 11.1%, *p* = 0.365); N/V (8.9% vs. 7.4%, *p* = 0.615)
	135			52.6%	16.3%	M39.3	38.5%	19.3%			
			**<0.01**			**<0.01**					

Abbreviations: CRT, chemoradiation; CT, chemotherapy; DFS, disease-free survival; OS, overall survival; LRR, locoregional recurrence rate; DM, distant metastases; N/V, nausea and vomiting; GI, gastrointestinal; NS, not significant; Cx, complication; CTx, chemotherapy; UVA, univariate analysis; MVA, multivariate analysis; LNR, lymph node ratio. Statistically significant *p*-values marked in bold font.

**Table 3:** Pooled results of endpoints.

Studies	No. of Studies	No. of Patients	Heterogeneity *p*	I^2^ (%)	Heterogeneity	Pooled Results (95% CI)	*p* (Pooled Analyses)	Egger’s *p*	
DFS, controlled comparisons									
All studies	11	2379	0.1	37.5%	Medium	OR 1.264 (0.997–1.603)	0.053	0.942	
RCTs	6	1089	0.964	~0.0%	Very low	OR 1.440 (1.110–1.867)	0.006	0.420	
Balanced	9	1913	0.61	~0.0%	Very low	OR 1.417 (1.171–1.715)	<0.001	0.824	
3-year DFS, single-arm analysis									
CRT arm	11	1225	<0.001	84.9%	Very high	62.5% (54.6–69.8)	NA	0.749	
CT arm	11	1154	<0.001	91.3%	Very high	57.1% (46.1–67.5)	NA	0.868	
5-year DFS, single-arm analysis									
CRT arm	8	848	<0.001	91.1%	Very high	53.3% (40.9–65.4)	NA	0.851	
CT arm	8	861	<0.001	93.4%	Very high	40.9% (27.3–56.0)	NA	0.748	
OS, controlled comparisons									
All studies	13	2583	0.06	41.2%	Medium	OR 1.124 (0.881–1.434)	0.347	0.760	
RCTs	6	1089	0.406	1.5%	Very low	OR 1.208 (0.921–1.584)	0.172	0.622	
Balanced	9	1913	0.166	31.6%	Medium	OR 1.279 (0.995–1.644)	0.055	0.840	trimmed value ^†^
Stage ≥III	5	563	0.662	~0.0%	Very low	OR 1.663 (1.170–2.363)	0.005	0.023	OR 1.963 (1.443–2.671)
3-year OS, single-arm analysis									
CRT arm	11	1235	<0.001	90.8%	Very high	61.4% (51.1–70.8)	NA	0.479	
CT arm	11	1208	<0.001	95.0%	Very high	59.1% (44.4–72.3)	NA	0.513	
5-year OS, single-arm analysis									
CRT arm	8	848	<0.001	89.1%	Very high	55.8% (44.5–66.5)	NA	0.885	
CT arm	8	861	<0.001	93.9%	Very high	46.9% (32.0–62.4)	NA	0.922	
Locoregional recurrence, controlled comparison									
All studies	8	1724	0.073	46.1%	Medium	OR 0.559 (0.355–0.879)	0.012	0.439	
RCTs	4	960	0.569	~0.0%	Very low	OR 0.495 (0.342–0.715)	<0.001	0.307	
Balanced	6	1592	0.357	9.2%	Low	OR 0.472 (0.334–0.667)	<0.001	0.863	
Locoregional recurrence, single-arm analysis									
CRT arm	8	810	0.001	70.4%	High	11.3% (7.5–16.8)	NA	0.786	
CT arm	8	914	<0.001	73.7%	High	18.1% (13.1–24.4)	NA	0.459	
Distant metastasis, controlled comparison									
All studies	8	1724	0.504	~0.0	Very low	OR 0.768 (0.611–0.965)	0.023	0.004	OR 0.837 (0.655–1.070)
RCTs	4	960	0.511	~0.0	Very low	OR 0.788 (0.589–1.054)	0.108	0.027	OR 0.814 (0.612–1.083)
Balanced	6	1592	0.543	~0.0	Very low	OR 0.802 (0.634–1.015)	0.066	0.051	OR 0.908 (0.710–1.160)
Distant metastasis, single-arm analysis									
CRT arm	8	810	<0.001	74.3%	High	24.1% (18.1–31.2)	NA	0.621	
CT arm	8	914	<0.001	75.3%	High	30.9% (24.4–38.3)	NA	0.069	29.0% (22.6–36.3)
Complication of grade ≥3									
Leukopenia	8	1821	0.433	~0.0	Very low	OR 1.387 (1.109–1.734)	0.004	0.946	
N/V	7	1731	0.406	2.5%	Very low	OR 1.224 (0.893–1.677)	0.209	0.617	
Leukopenia, single-arm analysis									
CRT arm	10	1154	<0.001	91.9%	Very high	26.4% (17.7–37.5)	NA	0.105	
CT arm	10	1133	<0.001	91.1%	Very high	15.7% (9.4–25.0)	NA	0.074	17.1% (10.7–26.2)
N/V, single-arm analysis									
CRT arm	9	1108	<0.001	72.6%	High	11.2% (7.8–15.8)	NA	0.200	
CT arm	9	1089	0.005	63..8%	High	11.1% (7.6–16.0)	NA	0.014	11.7% (7.7–17.5)

Abbreviations: CI, confidence interval; OR, odds ratio; RCT, randomized controlled trial; CRT, chemoradiation; CT, chemotherapy; NA, not assessable; DFS, disease-free survival; OS, overall survival; N/V, nausea and/or vomiting. ^†^ Values from Duval and Tweedie’s trim and fill method.

## Supplementary Materials

The following are available online at https://www.mdpi.com/2072-6694/12/8/2125/s1, Table S1: Study quality evaluation sheet.
