# Supplementary material for: Oncologic Benefit of Adjuvant Chemoradiation after D2 Gastrectomy: A Stepwise Hierarchical Pooled Analysis and Systematic Review
Source: Cancers (Basel). 2020 Jul 31;12(8):2125. doi: 10.3390/cancers12082125 (PMC7465129; doi:10.3390/cancers12082125)
Supplement: Supplementary file 1 [file cancers-12-02125-s001.pdf]

Supplementary Materials: Oncologic Benefit of Adjuvant Chemoradiation after D2 Gastrectomy: A Stepwise Hierarchical Pooled Analysis and Systematic Review

Chai Hong Rim, In-Soo Shin, Hye Yoon Lee, Won Sup Yoon and Sunmin Park

Table S1. Scoring sheet according to Newcastle-Ottawa scle.

Author	Selection				Comparability	Outcome			Overall Score (9 to Be Full)
	1	2	3	4	1	1	2	3	
	Representativeness of the exposed cohort	Selection of the non exposed cohort	Ascertainment of exposure	Outcome of interest was not present at start of study	Comparability of cohorts on the basis of the design or analysis	Assessment of outcome	Was follow-up long enough for outcomes to occur	Adequacy of follow up of cohorts	
Markelis	1	1	1	1	0	1	0	0	5
Kwon	1	1	1	1	1	1	1	1	8
Zhu	1	1	1	1	2	1	1	1	9
Kim	1	1	1	1	2	1	1	1	9
Park	1	1	1	1	2	1	1	1	9
Kilic	1	1	1	1	0	1	0	1	6
Lee	1	1	1	1	0	1	1	1	7
Cao	1	1	1	1	2	1	1	1	9
Turanli	1	1	1	1	1	1	1	1	8
Yu	1	1	1	1	2	1	1	1	9
Peng	1	1	1	1	1	1	1	1	8
Li	1	1	1	1	1	1	1	1	8
Ma	1	1	1	1	1	1	1	1	8

© 2020 by the authors. Licensee MDPI, Basel, Switzerland. This article is an open access article distributed under the terms and conditions of the Creative Commons Attribution (CC BY) license (http://creativecommons.org/licenses/by/4.0/).
